# Genetic modulation of CWD prion propagation in cervid PrP *Drosophila*

**DOI:** 10.1042/BCJ20230247

**Published:** 2023-10-04

**Authors:** Alana M. Thackray, Erin E. McNulty, Amy V. Nalls, Alzbeta Cardova, Linh Tran, Glenn Telling, Sylvie L. Benestad, Sabine Gilch, Candace K. Mathiason, Raymond Bujdoso

**Affiliations:** 1 Department of Veterinary Medicine, University of Cambridge, Madingley Road, Cambridge CB3 0ES, U.K.; 2 Prion Research Center (PRC) and the Department of Microbiology, Immunology and Pathology, Colorado State University, Fort Collins, CO, U.S.A.; 3 Department of Biohazard and Pathology, WOAH Reference Laboratory for CWD (SLB), National Veterinary Institute, Postboks 64, 1431 Ås, Norway; 4 Faculty of Veterinary Medicine, University of Calgary, 3330 Hospital Drive NW, Calgary, AB T2N 4N1, Canada

**Keywords:** cervid, CWD, *Drosophila melanogaster*, infectivity, neurodegeneration, prion

## Abstract

Chronic wasting disease is a fatal prion condition of cervids such as deer, elk, moose and reindeer. Secretion and excretion of prion infectivity from North American cervids with this condition causes environmental contamination and subsequent efficient lateral transmission in free-ranging and farmed cervids. Variants of cervid PrP exist that affect host susceptibility to chronic wasting disease. Cervid breeding programmes aimed at increasing the frequency of PrP variants associated with resistance to chronic wasting disease may reduce the burden of this condition in animals and lower the risk of zoonotic disease. This strategy requires a relatively rapid and economically viable model system to characterise and support selection of prion disease-modifying cervid PrP variants. Here, we generated cervid PrP transgenic *Drosophila* to fulfil this purpose. We have generated *Drosophila* transgenic for S138 wild type cervid PrP, or the N138 variant associated with resistance to chronic wasting disease. We show that cervid PrP *Drosophila* accumulate bona fide prion infectivity after exposure to cervid prions. Furthermore, S138 and N138 PrP fly lines are susceptible to cervid prion isolates from either North America or Europe when assessed phenotypically by accelerated loss of locomotor ability or survival, or biochemically by accumulation of prion seeding activity. However, after exposure to European reindeer prions, N138 PrP *Drosophila* accumulated prion seeding activity with slower kinetics than the S138 fly line. These novel data show that prion susceptibility characteristics of cervid PrP variants are maintained when expressed in *Drosophila*, which highlights this novel invertebrate host in modelling chronic wasting disease.

## Introduction

Prion diseases are fatal transmissible neurodegenerative conditions of mammalian species that include Creutzfeldt–Jakob disease (CJD) of humans, bovine spongiform encephalopathy (BSE) of cattle, scrapie of sheep and chronic wasting disease (CWD) of cervids [1]. These conditions are characterised by the conversion of the normal host protein PrPC into the disease-associated conformer PrPSc that accumulates in the CNS of affected individuals [2], and on occasion in peripheral tissues and body fluids. The prion hypothesis proposes that the transmissible prion agent comprises principally, if not solely, of PrPSc [3]. Mammalian prions can exist as different strains of the transmissible agent that directly influence host range and the clinico-pathological features of prion disease in the affected host [1]. According to the prion hypothesis, prion strain-specific properties are dictated by the conformational arrangement of PrPSc [4–6]. Changes in the replication environment of prions can induce mutational change in their strain properties [7], an event that may occur during inter-species prion transmission where PrP primary structures differ [8,9]. In this context, prion propagation is considered to be responsible for the neurotoxicity seen in these diseases [10,11]. Prion diseases are transmissible within and between different species [1]. Animal prion diseases are therefore a zoonotic threat to humans through consumption of prion contaminated animal products. This threat has been realised with the epizootic of BSE in cattle and subsequent emergence of variant CJD (vCJD) in humans [12,13]. Consequently, there is a heightened interest in the monitoring and management of animal prion diseases in order to protect human health.

CWD is a contagious prion disease of cervid species including deer, elk, moose and reindeer [14–16]. Clinically affected animals are characterised by a wasting syndrome together with hypersalivation ataxia, and polyuria. During CWD progression, prion infectivity accumulates in the brain and also within the lymphatic system, intestinal tract, muscle, blood and additionally, in urine, saliva and faeces [17–20]. Because of peripheral distribution, CWD prions are secreted and excreted from affected animals. This leads to environmental prion contamination and subsequent efficient lateral transmission in free-ranging and farmed cervids. CWD is highly prevalent in North America and has been detected in 29 states of the USA and four Canadian provinces [21]. High levels of transmission occur in high-prevalence areas that have resulted in a significant decline in wild cervid populations, such as white-tailed deer and mule deer [22,23]. CWD has recently been described in Europe [24] with cases identified in free-ranging reindeer, moose and red deer in Finland, Norway and Sweden, collectively. Prion strain typing studies have reported no etiological link between North American and European CWD isolates [25,26]. The high level of CWD transmission in cervids has led to concerns about the spread of the disease to other animal species including humans. North American CWD isolates have been successfully transmitted experimentally to farmed animals including cattle, pigs and sheep [27–29]. The zoonotic potential of CWD has been examined by *in vivo* transmission studies in non-human primates and human PrP transgenic mice but remains unresolved [30].

In cervids, species-specific polymorphisms in PrP have been identified that correlate with CWD prevalence and disease progression [31–34]. For example, an S138N PrP dimorphism is found in fallow deer and American reindeer, where the N138 PrP allele is associated with reduced susceptibility to natural CWD infection N138 homozygous fallow deer are resistant to natural CWD infection [35,36], although these animals are susceptible to the disease following experimental intracerebral inoculation [37]. In North American reindeer, animals with S138 homozygosity developed prion disease after oral exposure to CWD prions whereas those with at least one N138 allele showed relative resistance [38,39]. In elk, genetic analysis identified an M132L dimorphism whereby M132 homozygosity was found to be more prevalent in CWD-positive free-ranging and farmed animals, which suggested the L132 variant was associated with relative resistance and delayed onset of disease [40,41]. Similarly, an S225F dimorphism has been identified in mule deer PrP with S225 homozygosity more prevalent in CWD-positive animals than the SF heterozygote genotype, suggesting the F225 variant was linked to reduced disease prevalence, protracted disease time course, and variations in disease pathology [42,43]. Different variants of white-tailed deer PrP exist that appear to modulate CWD susceptibility and include 95H (Q95H), 96G, 96S (G96S), 116G (A116G) and 226K (Q226K) [31–34]. These variants are caused by non-synonymous single-nucleotide polymorphisms (SNPs) in the cervid *PRNP* gene, and only one of these SNPs can be present in a haplotype. Homozygosity for the 96G variant makes deer highly susceptible to CWD, while other combinations of variants, including those heterozygous at position 96 (96GS), cause reduced susceptibility and increased disease durations [44–46].

The existence of cervid PrP variants with associated resistance to CWD has provided support for the development of cervid breeding programmes to enhance the distribution of these alleles in susceptible cervid populations [47]. It is envisioned this strategy will reduce the burden of CWD in cervids and therefore lower the zoonotic potential of this animal prion disease. An important requisite in achieving this goal is the development of a suitable experimental system that can characterise and support selection of potential CWD resistant cervid PrP genes more rapidly than can be assessed in the natural host. The invertebrate species *Drosophila melanogaster* is a suitable candidate for this purpose. *Drosophila* have emerged as a model system to study mammalian neurodegenerative diseases [48,49] and constitute a genetically well-defined organism that lend themselves to tractable transgenesis procedures for the introduction of exogenous genes into their genome [50] and their subsequent expression [51]. Significantly, we have previously reported that *Drosophila* transgenic for mammalian PrP are susceptible to mammalian prions as evidenced by a neurotoxic phenotype, the severity of which correlates with the level of accumulation of prion seeding activity and bona fide transmissible prion infectivity [52]. These novel studies have established PrP transgenic *Drosophila* as a suitable host to study mammalian prion disease biology.

The objective of our study presented here was to generate a new CWD prion infectivity bioassay, one that is relatively rapid and economically viable compared with the natural host, to support selection of prion disease-modifying cervid PrP variants. In doing so, we report for the first time the successful use of *Drosophila* as a new experimental system in the study of cervid PrP variants and their associated CWD susceptibility. We have achieved this with remarkable success through our demonstration that *Drosophila,* a normally PrP null host, made transgenic for a single gene, namely one that encodes cervid PrP, can propagate cervid prions. To do so, we have generated *Drosophila* transgenic for S138 wild type cervid PrP, or the N138 variant which is associated with resistance to CWD in cervids and performed prion infectivity studies in these novel fly lines. We have established that cervid PrP *Drosophila* are susceptible to cervid-derived CWD prion inocula and accumulate bona fide infectious prions that can infect a mammalian host, namely cervid PrP transgenic mice. We show that both S138 and N138 cervid PrP fly lines are susceptible to cervid prion isolates from either North America or Europe when assessed phenotypically by accelerated loss of locomotor ability or survival, or biochemically by accumulation of prion seeding activity. Significantly, after exposure to European reindeer CWD prions, N138 cervid PrP *Drosophila* accumulated prion seeding activity with reduced kinetics compared with the S138 fly line. These novel data show that prion susceptibility characteristics of cervid PrP variants are maintained when expressed in *Drosophila*. Collectively, these observations highlight the utility of cervid PrP *Drosophila* as a new experimental model for the characterisation of CWD resistant cervid PrP genes.

## Results

### Generation of cervid PrP *Drosophila*

**Figure 1.**
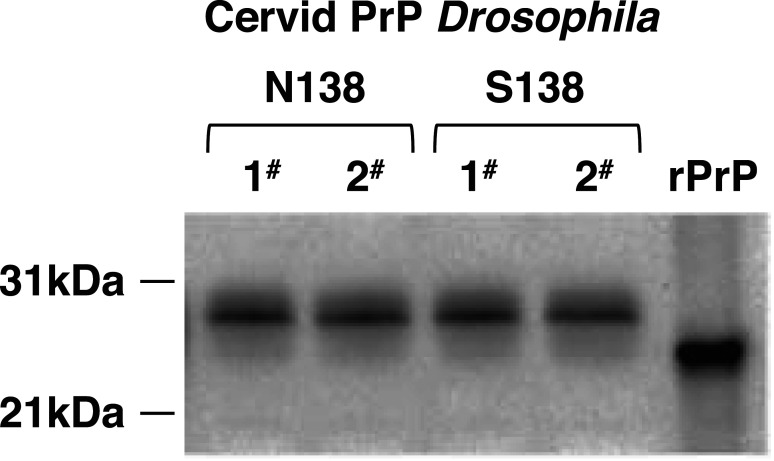
Western blot detection of prion protein expression in cervid PrP *Drosophila*. *Elav* x S138 (S138) and *Elav* x N138 (N138) white-tailed deer PrP *Drosophila* were harvested at 5 days of age. Fly head homogenates were prepared and analysed by SDS–PAGE and western blot with anti-PrP monoclonal antibody Sha31. The equivalent of five fly heads were run per track. Molecular mass marker values (kDa) are shown on the left-hand side. Ovine recombinant PrP (rPrP) was included as a positive control for the western blot. Numbers 1# and 2# represent duplicate fly lines of each cervid PrP genotype.

We generated *Drosophila* transgenic for variants of cervid PrP through the site-specific pUASTattB/PhiC31 integrase system, which delivers a single copy of the transgene of interest into a defined landing pad in the fly genome [50]. The PrP transgenes comprised DNA that encoded S138 (wild type) or N138 mature-length white-tailed deer PrP (amino acid residues 25–233), flanked by the coding sequences for an insect leader sequence peptide [53] at the 5′ end and the cervid glycosylphosphatidyl inositol (GPI) anchor sequence at the 3′ end. The PrP constructs were ligated into the *Drosophila* integration vector pUASTattB for injection into embryos of the 51D fly line. Stable balanced *UAS-*cervid PrP transgenic *Drosophila* were generated by conventional fly crossing and crossed with the *elav*-GAL4 driver fly line to induce pan neuronal PrP expression. Western blot analysis in Figure 1 showed that the cervid PrP variants expressed in *Drosophila* had a molecular mass of 29–31 kDa, like that of other species forms of PrP expressed in *Drosophila* [54]. Two replicate fly lines were prepared for each cervid PrP genotype to demonstrate the reproducibility of the single landing site transgenesis system and both sets of replicate fly lines showed similar PrP expression levels. N138 and S138 cervid PrP fly lines labelled 1# were used in this manuscript.

### Cervid CWD isolates

**Figure 2.**
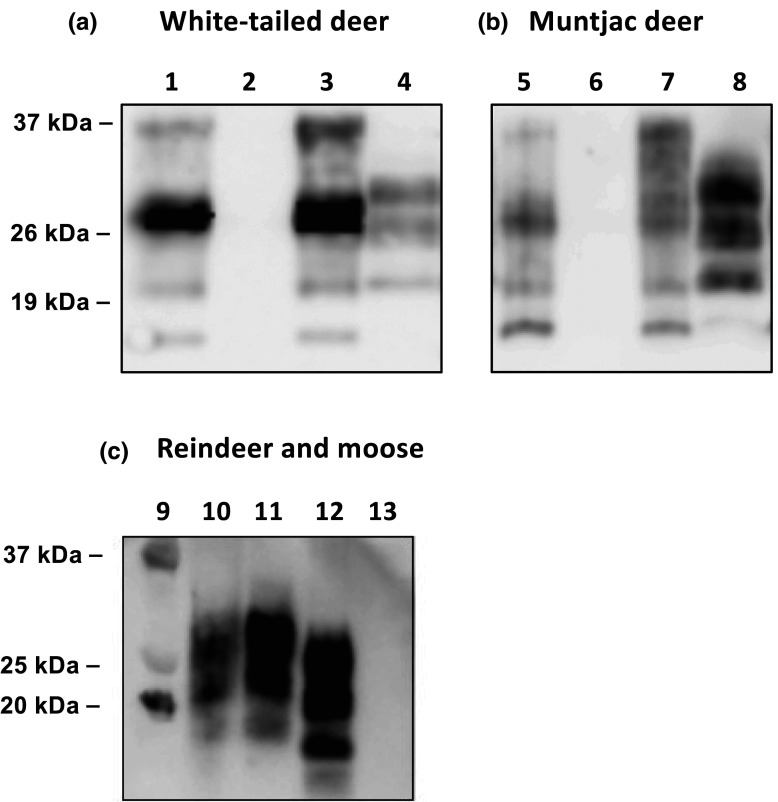
Western blot detection of Proteinase K-resistant PrPSc in North American and European CWD isolates. Brain homogenates prepared from white-tailed deer (**a**) or muntjac deer (**b**) without prion disease (lanes 1, 2, 5 and 6) or with experimental CWD (lanes 3, 4, 7 and 8) were assessed for Proteinase K-resistant PrPSc. Lanes 1, 3, 5 and 7 no Proteinase K digest; lanes 2, 4, 6 and 8 with Proteinase K digest. Homogenates from naturally infected reindeer parotid lymph node or brain or moose brain or negative control cervid tissue (**c**) were all treated with Proteinase K. Protein standard (lane 9); Reindeer 17-CD 11087 parotid lymph node (lane 10); Reindeer 17-CD 11087 brain (lane 11); moose 17-CD 11399 brain (lane 12); negative control cervid tissue (lane 13). Molecular mass marker values (kDa) are shown on the left-hand side.

The susceptibility of cervid PrP *Drosophila* to cervid prions was assessed with inoculum prepared from brain homogenate of CWD-experimentally challenged white-tailed deer or muntjac deer (North American samples) [55,56], or European naturally prion-infected reindeer (17-CD 11087) or moose (17-CD 11399). Inoculum was prepared from brain or parotid lymph node material in the case of reindeer 17-CD 11087. Prion-free brain material from white-tailed deer was used as control inoculum. The data in Figure 2a demonstrate PK sensitive (lane 2) and resistant (lane 4) cervid PrP present in brain tissue harvested from experimentally inoculated white-tailed deer (WTD) and the data in Figure 2b demonstrate PK sensitive (lane 6) and resistant (lane 8) cervid PrP present in brain tissue harvested from experimentally-inoculated muntjac deer of known CWD status [55,56] that was used here. The data in Figure 2c demonstrate PK resistant (lanes 10, 11 and 12) cervid PrP from naturally prion-infected reindeer or moose.

### Susceptibility of cervid PrP *Drosophila* to cervid prions

**Figure 3.**
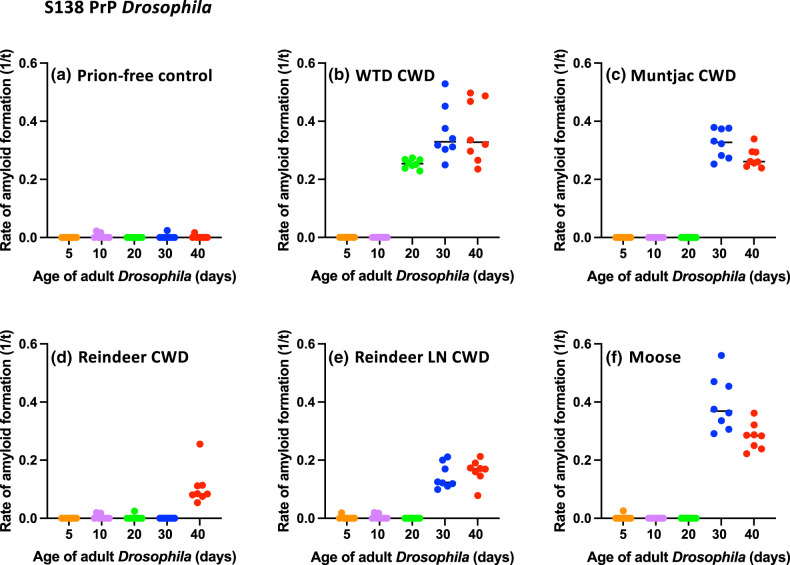

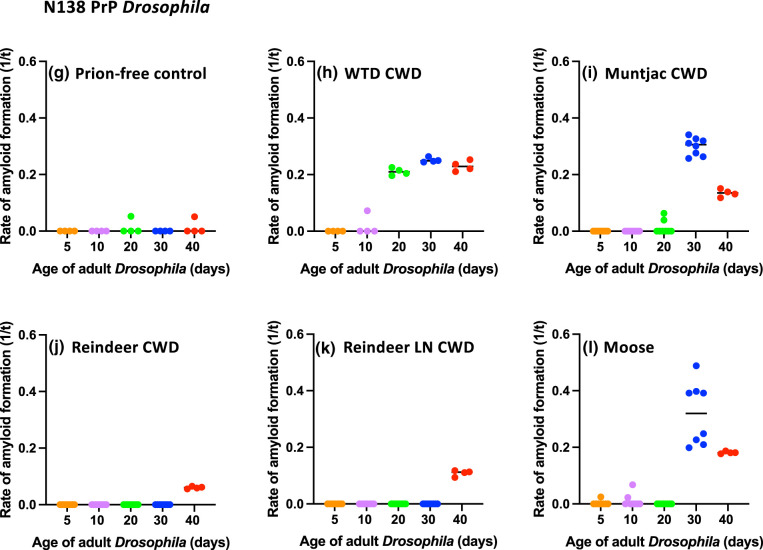
Prion seeding activity accumulation in CWD prion-exposed cervid PrP *Drosophila*. *Elav* x S138 (upper panel: **a**–**f**) and *Elav* x N138 (lower panel: **g**–**l**) cervid PrP *Drosophila* were exposed at the larval stage to experimental CWD-infected (**b**, **c**, **h**, **i**) or control prion-free cervid brain homogenate (**a** and **g**) or natural CWD-infected cervid parotid lymph node (**e** and **k**) or brain homogenate (**d**, **f**, **j** and **l**) at the larval stage. Adult *Drosophila* were collected at the indicated time points after hatching and head homogenate was prepared and used as seed in RT-QuIC reactions. Known RT-QuIC positive and negative fly head homogenates were included as controls for the assay. The data shown are rate of amyloid formation (1/*t*) for each treatment group.

*Drosophila* were exposed to prion-infected or prion-free control inoculum at the larval stage and after hatching were transferred to prion-free fly culture tubes. At various time points (≤40 days) during their adult lifespan, groups of *Drosophila* were euthanised, decapitated and homogenates were prepared from the isolated fly heads. These homogenates were used to seed *in vitro* RT-QuIC reactions in order to reveal cervid prion seeding activity. The data in Figure 3 show the level of prion seeding activity in adult white-tailed deer PrP *Drosophila*.

In S138 cervid PrP *Drosophila* (Figure 3a–f) base-line levels of prion seeding activity were seen in RT-QuIC reactions seeded with fly head homogenate prepared from prion-free control inoculated cervid PrP *Drosophila* (Figure 3a). In contrast, significant levels of prion seeding activity were detected in S138 cervid PrP *Drosophila* aged ≥20 days of age, after exposure to experimental CWD from white-tailed deer (Figure 3b). Prion seeding activity was also detected in S138 cervid PrP *Drosophila* aged ≥30 days of age, after exposure to experimental CWD from muntjac deer (Figure 3c) and from naturally infected European reindeer (Figure 3d, brain inoculum and Figure 3e, lymph node inoculum) or moose (Figure 3f).

Similar trends were seen in N138 cervid PrP *Drosophila* (Figure 3g–l) in that base-line levels of prion seeding activity were seen in RT-QuIC reactions seeded with fly head homogenate prepared from prion-free control inoculated cervid PrP *Drosophila* (Figure 3g). However, prion seeding activity was detected in N138 cervid PrP *Drosophila* aged ≥20 days of age, after exposure to experimental CWD from white-tailed deer (Figure 3h). Prion seeding activity was also detected in N138 cervid PrP *Drosophila* aged ≥30 days of age, after exposure to experimental CWD from muntjac deer (Figure 3i). Following exposure to naturally infected European reindeer (Figure 3j, brain inoculum and Figure 3k, lymph node inoculum), prion seeding activity was only detected at low levels in N138 cervid PrP *Drosophila* at 40 days of age. Prion seeding activity was detected in N138 cervid PrP *Drosophila* aged ≥30 days of age after exposure to naturally infected European moose brain (Figure 3l). The accumulation of prion seeding activity in CWD-exposed S138 or N138 cervid PrP *Drosophila* aged >10 days post hatching, following prion exposure at the larval stage, was indicative of accumulation of a replicating prion moiety in these flies.

### Accumulation of transmissible CWD prion infectivity in cervid PrP *Drosophila*

**Figure 4.**
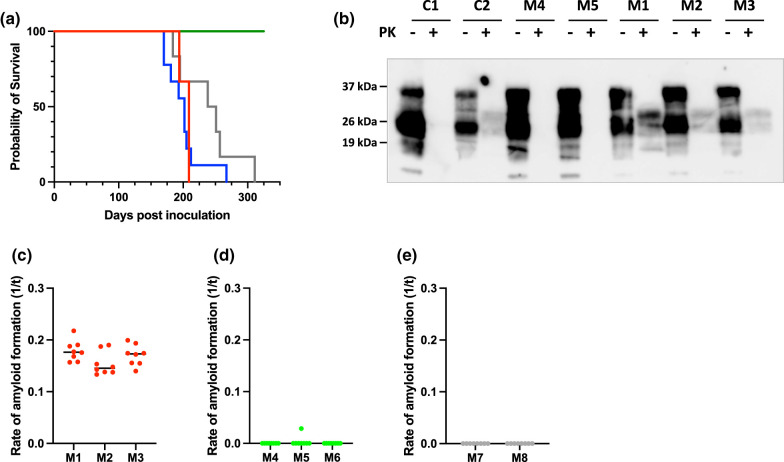
Transmissible CWD prion replication in cervid PrP *Drosophila*. *Elav* x N138 cervid PrP and *Elav* x 51D *Drosophila* were exposed to white-tailed deer experimental CWD-infected or prion-free brain homogenate at the larval stage. Head homogenates were prepared from adult flies aged 40 days and inoculated into Tg(CerPrP-E226)5037^+/−^ mice. (Negative control 51D *Drosophila* exposed to white-tailed deer prion-free brain homogenates were not assessed). (**a**) Survival times of mice inoculated with head homogenate from CWD-exposed *Elav* x N138 cervid PrP *Drosophila*. Control inoculated mice showed 100% (*N* = 2), with a survival time of 235 days. Control mice were euthanised 1 day after the last prion-inoculated mouse was euthanised due to clinical prion disease. Key — red: *Drosophila*; grey: white-tailed deer; blue: muntjac; green: control. (**b**) Western blot detection of Proteinase K-resistant PrP27-30 in the brains of prion diseased mice; Mouse sample numbers as follows: C1 uninoculated mouse (negative control); C2 cervid CWD inoculated mouse (positive control); M1–3 are mice inoculated with brain homogenate from *Elav* x N138 cervid PrP *Drosophila* exposed to cervid CWD inoculum; M4 and M5 are mice inoculated with brain homogenate from *Elav* x N138 cervid PrP *Drosophila* exposed to prion-free cervid brain homogenate. (**c**–**e**) RT-QuIC activity in the brains of mice inoculated with *Drosophila* head homogenate. Mouse sample numbers where relevant as in (**b**) above. M6 as for M4 and M5 in (**b**) above; M7 and M8 are mice inoculated with brain homogenate from *Elav* x 51D *Drosophila* exposed to cervid CWD brain homogenate. The dots in each panel refer to individual replicates of the RT-QuIC assay (*N* = 8).

We next investigated whether bona fide transmissible prions accumulated in CWD-exposed cervid PrP *Drosophila*. To do so, head homogenate prepared from 40-day-old *Drosophila* previously exposed to experimental CWD inoculum at the larval stage, was inoculated into Tg(CerPrP-E226)5037^+/−^ mice [57] that were subsequently assessed for the development of prion disease as shown by the data in Figure 4. Mice inoculated with CWD-exposed cervid PrP *Drosophila* head homogenate developed signs of clinical end-stage murine prion disease with a mean incubation time of 180 ± 5 days (Figure 4a). For comparison, Tg(CerPrP-E226)5037^+/−^ mice inoculated with brain material from CWD-positive white-tailed deer or muntjac showed incubation times of 239 ± 46 and 200 ± 29 days, respectively (Figure 4a and Table 1). The brains of mice with clinical end-stage murine prion disease inoculated with CWD-exposed cervid PrP *Drosophila* head homogenate contained Proteinase K-resistant PrP^27–30^ (Figure 4b) and RT-QuIC prion seeding activity (Figure 4c), which confirmed the presence of prion disease in these animals. The data in Figure 4 also show that in contrast, Tg(CerPrP-E226)5037^+/−^ mice inoculated with head homogenate from 40 day old cervid PrP *Drosophila* exposed at the larval stage to prion-free cervid brain homogenate, or 40 day old 51D *Drosophila* exposed at the larval stage to experimental CWD inoculum failed to develop murine prion disease (Figure 4b, d, e). Figure 4d demonstrated base-line levels of prion seeding activity. Collectively, these data show that cervid PrP *Drosophila* were susceptible to infection with cervid prions and in the process generated transmissible prions that caused bona fide prion disease in a mammalian host that expresses cervid PrPC.

**Table 1:** Transmission of CWD prions in cervid PrP mice

Inoculum	Cervid PrP mouse bioassay (Days post inoculation ± SD)
CWD^+^ cervid PrP *Drosophila*	204 ± 5
CWD^+^ white-tailed deer	239 ± 46^1^
CWD^+^ muntjac	200 ± 29^1^

Cervid PrP transgenic mice were inoculated with CWD material from cervid PrP *Drosophila*, white-tailed deer or muntjac. Days post inoculation represents the time between prion inoculation of the mice and their euthanasia at the onset of clinical prion disease.

1 Data from Nalls et al. [94] and [56].

### CWD prion-induced toxicity in cervid PrP *Drosophila*

It is well established that prion replication in mammalian hosts is associated with prion-induced toxicity [58,59]. To determine whether the replication of CWD prions in cervid PrP *Drosophila* was accompanied by a toxic phenotype in these flies we first performed a negative geotaxis climbing assay using adult *Drosophila* previously exposed to cervid prions at the larval stage. The locomotor activity of prion-exposed *Drosophila* was assessed over a 50-day period and expressed as a performance index [52].

**Figure 5.**
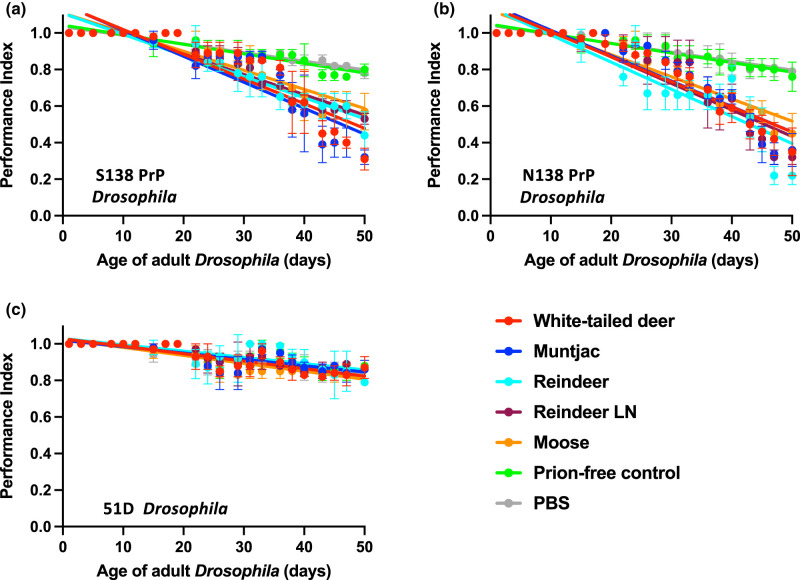
Accelerated decline in locomotor activity of CWD prion-exposed cervid PrP *Drosophila*. (**a**) *Elav* x S138 (S138); (**b**) *Elav* x N138 (N138) white-tailed deer PrP *Drosophila* and (**c**) *Elav* x 51D *Drosophila* were exposed to experimental CWD-positive or control prion-free cervid brain homogenate, or natural CWD-infected reindeer parotid lymph node or brain or moose brain homogenate or PBS at the larval stage. After hatching, flies were assessed for locomotor activity by a negative geotaxis climbing assay. The mean performance index is shown for three groups of *n* = 15 flies of each genotype per time point. Statistical analysis of the linear regression plots was performed using an unpaired (two-tailed) Student *t*-test. All CWD prion-exposed S138 and N138 PrP *Drosophila* treatment group plots were significantly different (*P* < 0.05) from their respective prion-free control plots over the whole of the climbing assay time course, except S138 PrP *Drosophila* exposed to WTD CWD or moose CWD, which were significantly different (*P* < 0.05) from the prion-free control group over days 10–50 and 17–50, respectively.

The data in Figure 5 show that adult S138 (Figure 5a) and N138 (Figure 5b) cervid PrP *Drosophila* developed a toxic phenotype after exposure to cervid prions evidenced by a significantly (*P* < 0.05) accelerated decrease in locomotor ability compared with flies of the same genotype exposed to prion-free control inoculum, which became progressively more severe with age. In contrast, negative control 51D *Drosophila* (Figure 5c) showed no difference in their locomotor ability following exposure to CWD prions or control inoculum.

**Figure 6.**
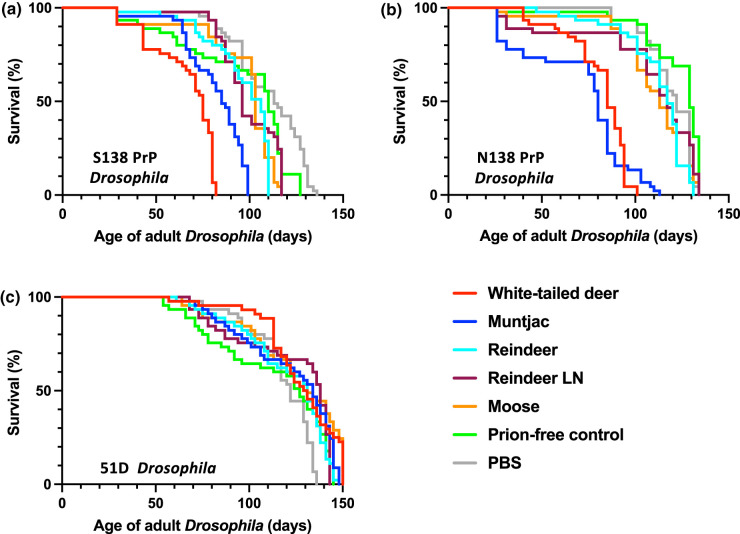
Accelerated loss of survival of CWD prion-exposed cervid PrP *Drosophila*. (**a**) *Elav* x S138 (S138); (**b**) *Elav* x N138 (N138) white-tailed deer PrP *Drosophila* and (**c**) *Elav* x 51D *Drosophila* were exposed to experimental CWD-positive or control prion-free cervid brain homogenate, or natural CWD-positive reindeer parotid lymph node or brain or moose brain homogenate or PBS at the larval stage. After hatching, the number of surviving flies was recorded three times a week and the data shown as Kaplan–Meier plots.

Clinical prion disease leads to a shortened life span in susceptible hosts [1,58]. Accordingly, we next investigated whether the toxic phenotype observed in cervid prion-exposed cervid PrP *Drosophila* affected survival of these flies. The data in Figure 6 show survival curves for adult cervid PrP *Drosophila* previously exposed to control or cervid prion inoculum at the larval stage. The median survival time for prion-free control exposed S138 (Figure 6a) and N138 (Figure 6b) adult cervid PrP *Drosophila* was 110 and 129 days, respectively (Table 2). In contrast, both S138 and N138 adult cervid PrP *Drosophila* showed a reduction in median survival time after exposure to cervid prions at the larval stage. The median survival times of S138 and N138 cervid PrP *Drosophila* were more reduced in response to North American CWD isolates (75–85 days) compared with the response seen with European CWD isolates (96–117 days). The median survival time for prion-free control exposed 51D adult *Drosophila* (Figure 6c) was 127 days (Table 2) and similar survival times were seen for this fly line after exposure to CWD prion inoculum.

**Table 2:** Median survival times of CWD prion-exposed cervid PrP *Drosophila*

Drosophila fly line	Median survival time (days)					
	Prion-free control	White-tailed deer	Muntjac	Reindeer	Reindeer LN	Moose
S138 PrP	110	75 (*P *< 0.0001)	85 (*P *< 0.0001)	106 (*P *= 0.0003)	96 (*P *> 0.05)	103 (*P *= 0.0038)
N138 PrP	129	85 (*P *< 0.0001)	80 (*P *< 0.0001)	117 (*P *< 0.0001)	117 (*P *= 0.0035)	113 (*P *< 0.0001)
51D	127	130 (*P *= 0.0165)	134 (*P *> 0.05)	131 (*P *> 0.05)	138 (*P *> 0.05)	131 (*P *= 0.0032)

*Elav* x S138 (S138), *Elav* x N138 (N138) white-tailed deer PrP *Drosophila* and *Elav* x 51D *Drosophila* were exposed to experimental CWD-positive or control prion-free cervid brain homogenate or natural CWD-infected reindeer parotid lymph node or brain homogenate or moose brain homogenate at the larval stage. After hatching, the number of surviving flies was recorded three times a week. Median survival times (in days) were obtained from Kaplan–Meier plots (as shown in Figure 6). Statistical analysis was performed by Log-rank (Mantel–Cox) test to compare survival curves of CWD prion-exposed treatment groups with the respective prion-free control group for each *Drosophila* genotype and *P*-values are shown in brackets.

Collectively, these data show that the toxic responses seen by cervid PrP *Drosophila* in response to CWD isolates are prion-mediated and PrP-dependant.

## Discussion

We generated *Drosophila* transgenic for S138 or N138 variants of white-tailed deer PrP by PhiC31 site-specific germ line transformation under expression control by the bi-partite GAL4/UAS system. Site-specific transgene insertion in the fly genome allowed us to test the hypothesis that single amino acid codon changes in cervid PrP modulate CWD prion-induced toxicity in *Drosophila*. Cervid PrP expressed in its natural mammalian host has a molecular mass of 30–35 kDa and comprises un-glycosylated and N-linked mono-, and di-glycosylated variants [60,61]. White-tailed deer PrP expressed in *Drosophila* had a molecular mass of ∼29–31 kDa and comprised two major protein bands in a similar manner seen for other mammalian species forms of prion protein expressed in the fly [62–66]. The lower molecular weight for mammalian PrP expressed in *Drosophila* compared with that seen in its natural host is likely to represent the differences in protein glycosylation between the invertebrate and mammalian species [67]. Although expression of PrP is required for the establishment of prion disease within a host, glycosylation of the protein is not necessary. Mice that only express un-glycosylated PrP can sustain prion disease, although different prion strains appear to have different requirements for each of the glycosylation sites of the host PrP [68]. As shown here, the S138N dimorphism in cervid PrP, a conserved change between two amino acids with polar neutral side chains, would appear to have no effect upon the expression of the prion protein in the fly since both variants showed a similar molecular profile and expression level.

We showed that S138 and N138 cervid PrP *Drosophila* were susceptible to CWD prion infectivity. We demonstrated that cervid PrP *Drosophila* exposed to CWD prions displayed cardinal features of mammalian prion disease that included progressive accumulation of prion seeding activity concomitant with increasing severity of a neurotoxic phenotype shown by an accelerated loss of locomotor ability and survival. Strikingly, head homogenate from CWD-exposed cervid PrP *Drosophila* induced prion disease in a mammalian host, namely mice transgenic for white-tailed deer PrP carrying the E226 genotype. These observations are compatible with authentic prion infection in CWD-exposed cervid PrP *Drosophila*. The CWD inocula we have used in our studies reported here included experimental isolates from North American white-tailed deer and muntjac [55,56] and natural isolates from one European moose and one reindeer. Although not formally quantified, cervid PrP *Drosophila* appeared to be more sensitive to CWD isolates from North America compared with those from Europe. This may reflect a higher titre of prion infectivity in experimental isolates compared with those from cervids with natural CWD infection. Alternatively, this may reflect a difference in response to different CWD prion strains since prion strain typing studies have shown no evidence for commonality in prion strains between North American and European CWD isolates [25]. Whatever the case, our observations show that cervid PrP *Drosophila* can detect a broad range of CWD isolates in a relatively rapid manner providing strong support for a new bioassay for mammalian prions. This is supported by our previous reports that show PrP transgenic *Drosophila* are permissive for authentic mammalian prion propagation [52] and are significantly more sensitive than PrP transgenic mice and PMCA for the detection of zoonotic mammalian prions. Here, we show that prion seeding activity was detected in adult cervid PrP *Drosophila* ≥20 days of age after exposure to CWD material at the larval stage. This shows that the cervid PrP transgenic *Drosophila* bioassay is ≈10× faster than the mouse prion bioassay. Furthermore, the tractable nature of *in vivo* studies with cervid PrP transgenic *Drosophila* provides a new experimental animal system to address important aspects of CWD prion propagation namely, the mechanism of CWD prion-induced neurodegeneration and the transcellular spread of CWD prions.

*Drosophila* do not normally express PrP and their genome does not contain an orthologue of the mammalian prion protein gene [69]. Our use of site-specific single gene-copy insertion [50] of S138 or N138 cervid PrP variants in the fly genome allowed us to test the hypothesis that individual amino acid codon changes in cervid PrP are directly responsible for the modulation of CWD prion-induced toxicity in *Drosophila*. Strikingly, while both S138 and N138 cervid PrP *Drosophila* were susceptible to CWD prions, we have found that following exposure to CWD inoculum from reindeer, N138 PrP *Drosophila* accumulated prion seeding activity with slower kinetics compared with that seen in the similarly treated S138 PrP fly line. Collectively, these observations appear to recapitulate findings in natural hosts, such as fallow deer and reindeer, where the N138 PrP variant is associated with a level of resistance to CWD [35]. However, the N138 PrP variant does not provide absolute resistance in cervids since fallow deer are susceptible to prion disease after intracerebral inoculation with CWD inoculum [37]. Furthermore, N138 homozygous reindeer inoculated orally with CWD prions succumb to clinical prion disease with shortened incubation times albeit with lower levels of PK-resistant PrPSc in their brains compared with reindeer that carry the S138 PrP allele [38,39]. In addition, knock-in transgenic mice homozygous or heterozygous for N138 cervid PrP develop subclinical prion disease when inoculated with CWD prions [70]. These mice display low levels of prion seeding activity in their brains compared with spleen tissue. These mice also have detectable prion seeding activity in faeces, which suggests that CWD could be transmitted from N138 PrP homozygous or heterozygous cervids that have subclinical infection. Our observations in *Drosophila* and those by others in natural cervid hosts suggest that N138 cervid PrP can show resistance to CWD prion-induced PrP misfolding, or that the resultant misfolded PrP is less resistant to metabolism compared with the wild type S138 variant. Distinguishing between these possibilities will be aided by structural comparisons between S138 and N138 cervid PrP using *in silico* molecular dynamics simulations and by their use as substrate for *in vitro* PMCA, as has been performed for other variants of cervid PrP [71,72]. The replication efficiency of CWD prions in PMCA is lower when brain homogenate from knock-in transgenic mice homozygous for N138, rather than S138, cervid PrP is used as substrate [70]. However, the replication efficiency is lower still when the substrate is brain homogenate from mice heterozygous for these two cervid PrP variants. The mature form of the mammalian prion protein is composed of a flexible N-terminal domain and a more structured C-terminal globular domain, with the latter composed of 3-alpha helices and a short two stranded antiparallel beta-sheet [73–75]. The polypeptide segment that links beta-strand-2 and helix-2 (beta-2 — alpha-helix-2 loop) plays an important role in the PrPC to PrPSc conversion [76,77]. It follows that amino acid changes that affect the antiparallel beta-sheet will impact upon the beta-2 — alpha-helix-2 loop. The S138N polymorphism is situated between beta-strand-1 and alpha-helix 1, and may well influence structural conversion of PrP via an impact on the antiparallel beta-sheet thereby accounting for its associated change in CWD susceptibility.

CWD is problematic amongst cervids because it is a contagious prion disease that is efficiently transmitted horizontally by direct contact [17] or through environmental exposure [78]. As a consequence, the management of CWD is challenging and control measures have typically relied upon quarantine coupled with herd reduction and depopulation, strategies that may be considered effective for farmed animals but less so for free-roaming cervids. The identification of cervid PrP variants associated with resistance to CWD has stimulated support for disease management by selective breeding programmes whereby the frequency of highly susceptible alleles is reduced and that of more resistant alleles is increased [31–34]. This type of approach has been used to reduce the prevalence of natural scrapie in sheep by the introduction of ovine PrP variants that were resistant to the condition [79,80]. Haley et al. [81] have described a selective breeding programme for captive white-tailed deer in a high-prevalence CWD-endemic area aimed at removal of the highly susceptible 96G *PRNP* variant (i.e. those variants without non-synonymous SNPs at other positions) in favour of the less susceptible 95H, 96S and 226K variants. The resultant decrease in frequency of the 96G variants and concomitant increase in 95H, 96S and 226K variants was accompanied by a successful decrease in prevalence of CWD. However, it has been shown, at least experimentally, that cervids harbouring the 95H, 96S and 226K PrP variants are susceptible to CWD infection, following experimental or natural exposure, albeit with a protracted disease progression. Furthermore, to date no cervid PrP variant has been identified that provides total resistance to CWD. Therefore, it is a realistic possibility that cervids harbouring the currently identified CWD resistant PrP variants that acquire the disease may act as ‘silent carriers’ and transmit prions to the environment for long periods of time [70]. It will be important therefore to study peripheral pathogenesis of CWD in cervids with resistant PrP variants. In this regard, cervid PrP *Drosophila* provide a suitably sensitive bioassay to assess the level of infectious prions that may be shed from ‘silent carriers.’

CWD can be transmitted to farm animal species, at least by experimental transmission [27–29]. This has raised concerns that CWD is a potential zoonotic for humans and a threat to public health. Currently, there is no epidemiological evidence to suggest a link exists between CWD in cervids and the increased prevalence of established prion diseases in humans, or the occurrence of new conditions analogous to the detection of vCJD in humans as a consequence of BSE in cattle [30]. Human PrP is capable of being converted to an abnormal form by CWD PrPSc *in vitro* amplification reactions [82–84]. *In vivo* bioassays using non-human primates and human PrP transgenic mice have been performed to model permeability of the human species barrier to CWD prions. Squirrel monkeys and possibly cynomolgus macaques have been shown to be susceptible to oral inoculation with CWD prions. One study has reported CWD transmission in human PrP mice. In contrast, other studies have reported conflicting data with respect to CWD transmission in cynomolgus macaques and human PrP mice and the zoonotic potential of CWD is presently undecided [30]. However, the current zoonotic assessments of CWD are based on studies that have utilised the existing repertoire of cervid prion strains that propagate within the present landscape of cervid PrP genotypes. It is established that different cervid prion strains propagate preferentially in cervids with distinct cervid PrP genotypes [85]. Consequently, selective cervid breeding programmes that alter the frequency of CWD susceptible and resistance PrP genes in different cervid species may well cause emergence, or predominance, of previously unseen cervid prion strains with new transmission characteristics including possible altered zoonotic potential. A precedent for this scenario was seen in sheep following ovine PrP breeding programmes aimed at the eradication of classical scrapie. While breeding for an increase in ovine PrP genotypes resistant to classical scrapie in flocks led to a decrease in the prevalence of this particular prion disease, it was associated with the emergence of previously unknown atypical scrapie [86]. Classical and atypical scrapie in sheep represent different ovine prion strains with different transmission properties. Furthermore, while classical scrapie has been considered non-pathogenic for humans [87,88], it is not clear if this is the case for atypical scrapie. For these reasons, it will be important to ensure that any implemented cervid PrP genotype breeding programme is associated with detailed monitoring of new CWD prion strains and a robust assessment of their zoonotic potential. The use of human PrP *Drosophila* could serve as a new bioassay host to help assess the zoonotic potential of CWD prion strains.

## Methods

### Generation of cervid PrP transgenic *Drosophila*

*Drosophila* transgenic for either S138 (wild type) or N138 white-tailed deer PrP were generated by pUASTattB/PhiC31-mediated site-specific transformation [50]. The cervid PrP transgenes comprised DNA encoding an insect secretion signal peptide at the 5′ end [53] followed by DNA encoding the S138 or N138 variants of mature-length white-tailed deer PrP (GenBank accession number AF156185, amino acid residues 25–233) and DNA encoding the cervid PrP GPI anchor signal sequence (amino acid residues 234–256) at the 3′ end. The cervid PrP transgenes were prepared by a two-step PCR as previously described [89]. The first PCR used plasmid DNA that contained cervid PrP DNA encoding either S138 or N138 white-tailed deer PrP as substrate and oligonucleotide primers CerPD1F: 5′ CCA TCT TCT GGC TGC TCA GAC CTT CGC CCA GAA GAA GCG ACC AAA ACC TG 3′ and CerPD1R: 5′ GTC CGC TCG AGT CTA GAC TAT CCT ACT ATG AGA AAA ATG 3′. A second PCR was carried out using the 765 bp product of the first PCR reaction as substrate and oligonucleotide primers PD2F: 5′-GGC GAA TTC ATG GCG AGC AAA GTC TCG ATC CTT CTC CTG CTA ACC GTC CAT CTT CTG C-3′ and CerPD1R already listed above. Site-specific transformation of the pUASTattB-PrP constructs into the RFP-free 51D variant fly line (y[1] M{vas-int.Dm}ZH-2A w[*]; M{3xP3-RFP.attP}ZH-51D) was performed by the Department of Genetics, Cambridge University. F1 flies were balanced, the inserted PrP transgenes verified by DNA sequence analysis, and viable fly lines w; M{Cer-PrP-S138(GPI).attP}ZH-51D and w; M{Cer-PrP-N138(GPI).attP}ZH-51D maintained as balanced stocks by conventional fly crosses. The following fly lines were obtained from the Department of Genetics, University of Cambridge, U.K. *Elav-GAL4* (P{w[+mW.hs]=GawB}elav[C155]), 51D (w; M{3xP3-RFP.attP}ZH-51D). All fly lines were raised on standard cornmeal media at 25°C, maintained at low to medium density. Flies were used in the assays described below or harvested at various time points and then frozen at −80°C until required.

### Preparation of *Drosophila* head homogenate

*Drosophila* head homogenates were prepared as previously described [89].

### Western blot

#### Cervid PrP *Drosophila*

Fly head homogenates (5 fly heads per track) were prepared for SDS–PAGE and western blot as described in detail previously [90] except that the nitrocellulose membranes were probed with a 1 : 2000 dilution of anti-PrP monoclonal antibody Sha31 [91].

#### North American cervid CWD isolates and Tg(CerPrP) 5037 mouse brains

Cervid brain homogenate of cerebral cortex tissue from confirmed cases of experimental CWD in white-tailed deer or muntjac deer [55,56] or brain tissue from Tg(CerPrP) 5037-inoculated mice [55] was prepared as a 10% (w/v) homogenate in 1× PBS and stored at 4°C until further analysis. Homogenates were treated with Proteinase K (PK; Invitrogen) at 50 µg/ml and analysed by SDS–PAGE and western blot as previously described [55,56].

#### European cervid CWD isolates

Homogenates were prepared from CWD prion-infected reindeer brain or parotid lymph node or from moose brain. Prion-free brain material from white-tailed deer was used as control inoculum. Western blots were carried out on PK treated samples using the TeSeE western blot (Bio-Rad).

### CWD prion inoculation of *Drosophila*

Prion inocula consisted of cervid brain homogenate, prepared in normal saline, of cerebral cortex tissue from confirmed cases of experimental CWD in white-tailed deer or muntjac [55,56] and natural CWD in reindeer and moose, or reindeer parotid lymph node tissue. Confirmed CWD-free cervid brain tissue or PBS was used as control inocula. *Drosophila* at the larval stage of development were exposed to CWD-infected homogenate, prion-free control cervid brain homogenate or PBS. Two hundred and fifty microlitres of 1% (w/v) of cervid brain homogenate or parotid lymph node, prepared in PBS pH 7.4, were added to the top of the cornmeal that contained third instar *Drosophila* larvae in three-inch plastic vials. Following eclosion (i.e. hatching) flies were transferred to fresh non-treated vials.

### *Drosophila* negative geotaxis climbing assay

The locomotor ability of flies was assessed in a negative geotaxis climbing assay initiated with 45 (3 × *n* = 15) age-matched, pre-mated female flies in each treatment group as previously described [92]. The mean performance index ± SD at individual time points for each treatment group was plotted as a regression line.

### *Drosophila* survival assay

Survival of *Drosophila* was assessed and recorded as previously described [89]. Survival curves are shown as Kaplan–Meier plots.

### Prion inoculation of cervid PrP transgenic mice

*Drosophila* head homogenates were prepared from 40 day old flies that had been exposed at the larval stage to white-tailed deer Cervid Brain Pool 6 (CBP6) CWD homogenate. Twenty microlitres of a 1% *Drosophila* head homogenate was injected intracerebrally into 4–6 week old Tg(CerPrP-E226)5037^+/−^ mice (*N* = 4) at Colorado State University (CSU). Animals were anesthetised by use of isoflurane inhalation (1–5%), prior to, and during, inoculation as approved by CSU Institutional Animal Care and Use Committee (IACUC). All mice were housed in accordance with CSU Laboratory Animal Resources and IACUC protocols within the Pathology research facility at CSU. Mice were monitored for the development of clinical signs of mouse prion disease and euthanised by CSU IACUC approved protocols using carbon dioxide 30–70% inhalation when signs of terminal disease appeared. Survival was measured as the time (in days) between inoculation and death. For each group of mice, survival times are presented as mean ± SD. Prion disease was confirmed by detection of PK resistant PrPSc via western blot and by the presence of RT-QuIC prion seeding activity.

### Detection of prion seeding activity by RT-QuIC

Real-time quaking-induced conversion (RT-QuIC) with syrian hamster recombinant PrP (rhaPrP: amino acids 90–231) as substrate was performed as described previously [55]. Seed was *Drosophila* head homogenate prepared as described above and diluted 1 : 10 in 0.1% SDS [93].

### Statistical analysis

Statistical analysis of the negative geotaxis climbing assay data was performed by the unpaired (two-tailed) Student's *t*-test. Statistical analysis of median survival times was carried out using Kaplan–Meier statistics and differences between them were analysed by the Log-rank (Mantel–Cox) test method. All statistical analyses were performed using Prism (GraphPad Software Inc, San Diego, U.S.A.).

## Data Availability

All data are included in this manuscript.

## Open Access

Open access for this article was enabled by the participation of University of Cambridge in an all-inclusive *Read & Publish* agreement with Portland Press and the Biochemical Society under a transformative agreement with JISC.

## Abbreviations

BSE: Bovine spongiform encephalopathy
CJD: Creutzfeldt–Jakob disease
CWD: Chronic wasting disease
GPI: glycosylphosphatidyl inositol
PK: Proteinase K
PrP: prion protein
PrPC: normal cellular PrP
PrPSc: abnormal disease-specific conformation of PrP
RT-QuIC: real-time quaking-induced conversion
vCJD: variant Creutzfeldt–Jakob disease
WTD: white-tailed deer
